# P-1078. Gentamicin-releasing polydopamine catheter coating to prevent urinary catheter-related infections

**DOI:** 10.1093/ofid/ofae631.1266

**Published:** 2025-01-29

**Authors:** Gillian Su, Hannah Karp, Michael Schulz, Jayasimha Rao, Elizabeth Nowak, Nammalwar Sriranganathan

**Affiliations:** Virginia Tech, Blacksburg, Virginia; Virginia Tech Carilion School of Medicine, Roanoke, Virginia; Virginia Tech, Blacksburg, Virginia; Carilion Clinic/Virginia Tech Carilion School of Medicine, Roanoke, Virginia; Carilion Clinic, Roanoke, Virginia; Virginia Polytechnic Institute and State University, Blacksburg, Virginia

## Abstract

**Background:**

Catheter-associated urinary infections (CAUTIs) are among the most common healthcare-associated infections in the United States. The risk of CAUTI corresponds to the duration of catheterization; prolonged catheter uses impacts patient morbidity, mortality, and healthcare costs. *Pseudomonas aeruginosa* is a leading cause of CAUTI and is particularly adept at forming surface-associated biofilms that are difficult to treat. In the present study, we developed a catheter coating using polydopamine (PD) to embed gentamicin on the surface.

Bacterial Viability of PAO1 when exposed to PD-gentamicin coating

**Figure:**
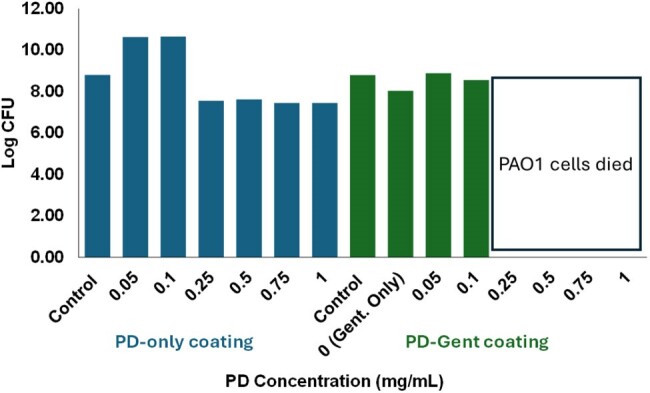

Colony-forming unit (CFU) count of PAO1 exposed to concentration series of PD-only and PD-Gent well plate coating.

**Methods:**

*Coating synthesis:* PD-antibacterial coating was synthesized using dopamine hydrochloride salt and antibiotic drugs (i.e., gentamicin) in an alkaline buffer in the presence of polyvinyl chloride (PVC) tubing. A similar procedure was used for the well-plate study using 150 µL of solution per well, followed by heating at 60 °C.

*Biofilm Quantification:* PAO1 was added to a 96-well plate or tubes and incubated statically for 24 h. Biofilms were stained with 0.1% crystal violet, solubilized with 33% glacial acetic acid, and measured at OD_590 nm_.

*Cell viability:* Colony forming units were measured using 10-fold serial dilutions on TSA agar.

Biofilm formation of PAO1 after incubation with PD-gentamicin coating in PVC tubes

**Figure:**
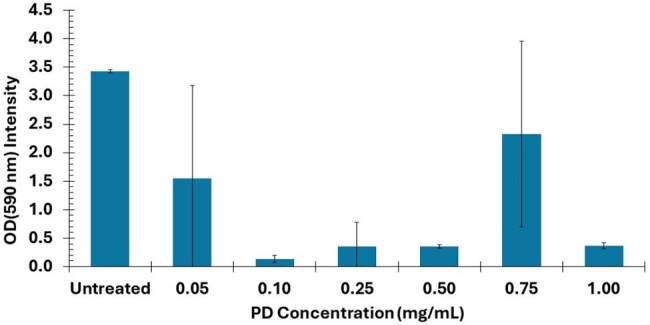

PAO1 biofilm concentration changes with changing the PD to gentamicin ratio during coating synthesis.

**Results:**

PD-gentamicin coated tubes and 96-well plates showed elimination of PAO1 viability at PD concentrations of 0.25 mg/mL and above but had little bactericidal effect at lower concentrations (Fig. 1). There was no reduction in viability in PAO1 exposed to PD or gentamicin alone. PAO1 biofilm was reduced in the median PD-gentamicin concentrations 0.1-0.5 mg/mL, but this effect was lost at the extremes of concentration (Fig. 2).

**Conclusion:**

The PD-gentamicin coating eliminated PAO1 viability and reduced biofilm at PD concentration 0.25 mg/mL. Similar results were observed in 96-well plates and PVC tubes, suggesting versatility in the adherence of the coating material. Additional studies are needed to assess the duration of these effects over time.

**Disclosures:**

**All Authors**: No reported disclosures

